# Fatty Acid Signatures in Different Tissues of Mediterranean Yellowtail, *Seriola dumerili* (Risso, 1810), Fed Diets Containing Different Levels of Vegetable and Fish Oils

**DOI:** 10.3390/ani10020198

**Published:** 2020-01-24

**Authors:** Francesco Bordignon, Ana Tomás-Vidal, Angela Trocino, Maria C. Milián Sorribes, Miguel Jover-Cerdá, Silvia Martínez-Llorens

**Affiliations:** 1 Department of Comparative Biomedicine and Food Science (BCA), University of Padova, Viale dell’Università 16, I-35020 Legnaro, Italy; francesco.bordignon.3@phd.unipd.it; 2 Institute of Animal Science and Technology, Group of Aquaculture and Biodiversity, Universitat Politècnica de València, Camino de Vera, 14, 46071 València, Spain; atomasv@dca.upv.es (A.T.-V.); mamisor@etsia.upv.es (M.C.M.S.); mjover@dca.upv.es (M.J.-C.); silmarll@dca.upv.es (S.M.-L.)

**Keywords:** brain, muscle, liver, greater amberjack, EPA, DHA

## Abstract

**Simple Summary:**

Most of the studies performed to date mainly investigated on the effects of dietary substitution of fish oil with vegetable oils on growth and fatty acid composition of edible muscle tissues. On the other hand, a few assessed how dietary lipids are retained in other tissues, such as brain, liver, and adipose tissue, which would provide further insights into the fatty acid requirements of new farmed marine fish species such as *Seriola dumerili*. Thus, this study evaluated how the replacement of fish oil with different proportions of vegetable oils in diets affects the tissue-specific fatty acid composition (also known as signature) of brain, muscle, liver, and visceral fat of *S. dumerili*. The fatty acid composition of the diet had a strong effect on the fatty acid signature of muscle, liver, and visceral fat, whereas the brain signature was less sensitive to dietary changes. These new insights contribute to identify the essential fatty acids requirements of Mediterranean yellowtail and to define the conditions under which the physiological functions in these fish are preserved when they are fed diets with low fish oil levels to guarantee the sustainability of their production and welfare.

**Abstract:**

The study aimed to evaluate how replacing different proportions of fish oil (FO) with vegetable oils (VO) in the diet of Mediterranean yellowtail, *Seriola dumerili* (Risso, 1810), affects the fatty acids (FA) signature, i.e.; overall FA profile, in different tissues. A total of 225 Mediterranean yellowtail juveniles (initial live weight: 176 ± 3.62 g) were fed for 109 days with one of three diets: A control diet (FO 100), with FO as the only lipid source, or diets with 75% and 100% of FO replaced with a VO mixture. At the end of the feeding trial, the brains, muscles, livers, and visceral fat were sampled in four fish per tank (12 per treatment), and their fat were extracted and used for FA analysis. The FA signatures of red and white muscle, liver, and visceral fat tissues changed when the dietary FA source changed, whereas FA signatures in the brain were rather robust to such dietary changes. These new insights might help evaluate whether key physiological functions are preserved when fish are fed diets with low FO levels, as well as define the dietary FA requirements of Mediterranean yellowtail to improve the sustainability of the production and welfare of the fish.

## 1. Introduction

The Mediterranean yellowtail, *Seriola dumerili* (Risso, 1810), also called the greater amberjack, is a new, high-value candidate for production in marine aquaculture. This cosmopolitan fish is mainly produced in Japan [1], Spain [2], Italy [3], and recently in Vietnam, Korea, and China [4]. It has high growth rates (reaching 6 kg within 2.5 years of culture), and exceptionally high consumer acceptance [4,5].

Since the Mediterranean yellowtail is an emerging species in aquaculture, its nutritional requirements have been defined in terms of major nutrients (47%–50% crude protein (CP) and 12%–14% crude lipid (CL)) [6,7,8,9,10]; however, little information is available on its fatty acid requirements [11], which thus require further investigation. Only one previous study assessed the effects of dietary fatty acids on Mediterranean yellowtail body composition [11].

Fatty acids (FA) play key roles in fish health and nutrition. They maintain the structural and functional integrity of cell membranes, provide metabolic energy through their oxidative metabolism, contribute to visual and brain development, and are precursors of a group of paracrine hormones with relevant biological roles known as eicosanoids [12,13,14]. Fish health, growth, and reproduction are strongly dependent on n-3 and n-6 polyunsaturated fatty acids (PUFA), especially arachidonic acid (AA, C20:4 n-6), eicosapentaenoic acid (EPA, C20:5 n-3), and docosahexaenoic acid (DHA, C22:6 n-3) [13,15,16,17]. However, marine carnivorous fish such as *S. dumerili* have limited ability to bio-convert the essential precursors of PUFA, such as linoleic acid (LA, C18:2 n-6) and alpha-linolenic acid (ALA, C18:3 n-3), into these essential FA [18,19].

The overall fatty acid profile within a given tissue, also known as its FA signature [20], is strongly dependent on the physiological function(s) of the tissue itself [21]. The liver and muscles are the main sites of β-oxidation [22], whereas the brain stores n-3 LC-PUFA, mainly DHA, which perform neurological functions [23,24,25]. Nevertheless, in nature, the FA signatures of prey tissues can be transferred to their predators with little modification and in a predictable manner [20,26,27]. Thus, in recent decades, FA have been extensively used as biomarkers in riverine and marine ecosystems [20,28,29]. Additionally, under farming conditions [30,31,32], the available literature collectively supports the conclusion that there is a close association between the diet and fillet FA composition in farmed fish, as reviewed by Turchini et al. [19].

As in other species [33], understanding the FA distributions and signatures within Mediterranean yellowtail tissues might help us understand whether the physiological needs and essential fatty acid (EFA) requirements of fish are satisfied under farming conditions. This is particularly crucial when diets that contain low levels of fish oil (FO) are used in fish farming. In fact, to ensure the long-term sustainability of the aquaculture sector and to improve its competitiveness, FO has been increasingly replaced by vegetable oils (VO) in fish diets because FO has a high cost and is available in finite and limited amounts [19]. Compared to FO, plant seed oils are rich in C:18 PUFA [19], but lacking in n-3 highly unsaturated fatty acids (HUFA), which are essential for marine fish [13].

Most studies performed in farmed fish to date have focused mainly on the effects of dietary lipids on the FA composition of edible muscle tissues [19]. Only one study [33], which investigated FA signatures in gilthead seabream (*Sparus aurata* L.; 1758), assessed how dietary lipids were retained in different tissues (brain, liver, and mesenteric adipose tissue) to obtain further insights into the FA requirements of farmed marine fish.

Therefore, this study was performed to evaluate how the replacement of FO with different proportions of VO in the diet affects the tissue-specific FA signatures and their robustness in the brain, muscle, liver, and visceral fat tissues of Mediterranean yellowtail (*S. dumerili*).

## 2. Materials and Methods

A feeding trial was performed at the Fish Nutrition Laboratory (LAC) of the Institute of Animal Science and Technology (ICTA) of the Universitat Politècnica de València (Polytechnic University of Valencia, Spain). The experimental protocol was approved by the Committee of Ethics and Animal Welfare of the Polytechnic University of Valencia, following the Spanish Royal Decree 53/2013 on the protection of animals used for scientific purposes.

The facility used a thermoregulated recirculating seawater system (65 m^3^ capacity), with a rotary drum-type filter and a mechanical gravity biofilter with a volume of 2 m^3^, equipped with 9 cylindrical fiberglass tanks with a capacity of 1750 L each with aeration.

### 2.1. Experimental Diets

Three isoproteic (59% CP, 50% digestible protein (DP)), and isolipidic (15% CL) extruded diets were formulated, with increasing levels of vegetable oils used to replace the fish oil in the diets as follows: 0% (control diet, FO 100), 75% (FO 25), and 100% (FO 0) replacement of FO with VO (Table 1). The mixture of vegetable oils used consisted of linseed oil, sunflower oil, and palm oil (4:3:3). Diets were prepared using a cooking-extrusion processor with a semi-industrial twin-screw extruder (CLEXTRAL BC-45; Firmity, St Etienne, France), at a screw speed of 100 rpm, temperature of 110 °C, and pressure of 40–50 atm, to obtain pellets 2–3 mm in diameter.

The FA profile of the experimental diets changed depending on the relative proportions of the two lipid sources included in them. Diets containing more FO contained higher proportions of highly unsaturated fatty acids from the n-3 series, whereas diets containing more vegetable oils had higher proportions of LA and ALA (Table 2). In all diets, C16:0 accounted for the bulk of the saturated fatty acids (SFA) present, C18:1n-9 for most of the monounsaturated fatty acids (MUFA), and EPA and ARA for most of the n-3 long-chain polyunsaturated fatty acids (PUFA). The inclusion of VO as a lipid source increased the dietary proportion of C18:1 n-9 (27.1%, 29.6%, and 32.7%), C18:1 n-6 (12.7%, 13.4%, and 14.9%), and ALA (14.1%, 14.3%, and 15.4%), whereas it decreased the proportions of total SFA, EPA, and docosahexaenoic acid (DHA) in the diets (SFA: 27.8%, 27.1%, and 25.5% in the FO 100, FO 25, and FO 0 diets, respectively; EPA: 5.81%, 4.34%, and 2.77%; DHA: 13.0%, 7.6%, and 4.3%) (Table 2).

### 2.2. Fish and Experimental Design

A total of 225 Mediterranean yellowtail juveniles obtained from a private hatchery (Futuna Blue S.A.; Cádiz, Spain) were transported to the Fish Nutrition Laboratory of the Universitat Politècnica de València for use in the feeding trial. After four weeks of acclimation, fish were individually weighed (initial weight: 176 ± 3.62 g), randomly distributed among nine tanks (25 fish per tank), and fed the experimental diets (FO 100, FO 25, or FO 0, with three tanks per diet) for 109 days.

During the trial, the water temperature averaged 21.5 ± 2.4 °C, the salinity was 31.5 ± 4.1 g L^−1^, and the dissolved oxygen content was 8.0 ± 0.4 mg L^−1^. The water pH ranged from 7.5 to 8.0, and the levels of nitrogenous compounds in the water were kept within the limits recommended for marine species.

### 2.3. In Vivo Recordings

Feed was distributed by hand, twice a day (at 09:00 and 16:00) for six days per week, until apparent satiation. Feed intake was recorded at each administration. Mortality was checked every day. At the beginning and at the end of the trial, fish were individually weighed after one day of feed deprivation and under light anaesthesia (10 mg L^−1^ clove oil containing 87% eugenol; Guinama^®^, Valencia, Spain). Fish health during weighing was assessed by direct observation.

After 109 days of feeding, four fish per tank (12 per treatment, 36 in total) were randomly sacrificed by lethal immersion in clove oil (150 mg L^−1^). Fish were dissected to sample their brain, white and red muscle, liver, and visceral fat tissues, which were then frozen in liquid nitrogen and stored at −80 °C until subsequent analyses.

### 2.4. Chemical Analyses of Diets and Fish Tissues

Diets were ground up and analysed according to AOAC procedures [34] to determine their dry matter (by heating at 105 °C until a constant weight was attained), ash (by incinerating them at 550 °C for 5 h), crude protein (AOAC official method 990.03, by the DUMAS direct combustion method, using a LECO CN628 apparatus, LECO, ST. Joseph, MI, USA), and crude lipid content (by extraction with methyl ether using an ANKOMXT10 extractor, ANKOM Technology, Macedon, NY, USA). Gross energy content (GE) was calculated according to Brouwer [35], from the C (g) and N (g) balance in the feed (GE = 51.8 × C − 19.4 × N). The carbon and nitrogen content were analysed based on the Dumas principle (TruSpec CN; Leco Corporation, St Joseph, MI, USA). All analyses were performed in triplicate.

After thawing, tissues were minced and homogenised. The crude fats in the brain, muscle, liver, and visceral fat tissue sampled were then extracted [36]. A direct method of fatty acid methyl ester (FAME) synthesis was used for this procedure [37]. The analysis of brain, muscle, liver, and visceral fat tissues was carried out using 10–30 mg of extracted crude fat from each tissue. First, 1 mL of tridecanoic acid (C13:0) was used as internal standard. Then, 0.7 mL of 10 N KOH and 5.3 mL of HPLC (high-performance liquid chromatography)-quality methanol were added. Tubes were incubated at 55 °C in a thermoblock for 1.5 h, and underwent vigorous shaking for 5 s every 20 min. After cooling at ambient temperature in a water bath, 1.5 mL of HPLC-quality hexane was added to the reaction tubes, which were then vortex-mixed and centrifuged at 1006× *g* for 5 min. After this, the hexane layer, containing the FAMEs, was placed into vials for analysis by gas chromatography. The vials were kept at −80 °C until gas chromatography was performed. The FAMEs were analysed in a Focus gas chromatograph (Thermo, Milan, Italy) equipped with a split/splitless injector and a flame ionisation detector. Separation of the methyl esters was performed in a SPTM 2560 fused silica capillary column (Supelco, PA, USA) (100 m × 0.25 mm × 0.2 μm film thickness). Helium was used as the carrier gas at a flow rate of 20 cm s^−1^. Samples were injected with a split ratio of 1:100.

The initial oven temperature, set at 140 °C, was held constant for 5 min, and then increased by 4 °C min^−1^ to 240 °C, at which this temperature was then maintained for 30 min. The FA were identified by comparing their retention times with those of the standards supplied by Supelco. The content of each type of FA was expressed as the percentage of the total FA content.

### 2.5. Statistical Analyses

The fish live weight, tissue fat content, and FA composition data were compared by analysis of variance (ANOVA), with diet included as the main effect and tank included as a random effect. The PROC MIXED procedure of the Statistical Analysis System (SAS) software [38] was used for all analyses. Adjusted means were compared among treatment levels using Bonferroni’s t-test. Differences between means with *p* ≤ 0.05 were considered statistically significant.

## 3. Results

**Figure 1:**
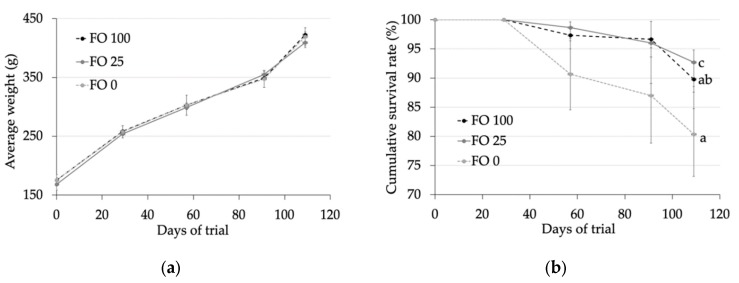
(**a**) Fish live weight (g) and (**b**) survival (%) of fish fed experimental diets during the 109 days of trial. Values are expressed as means ± standard error.

In the present study of *S. dumerili* juveniles (176 g of initial live weight), diets containing only VO as the lipid source had no effect on fish growth (Figure 1). In fact, fish fed FO 100, FO 25, and FO 0 diets reached a final weight of 423, 409, and 419 g, respectively (*p* > 0.05). The survival rate was lower in fish fed diets without fish oil compared with those in fish fed diets containing 100% (FO 100) or 25% (FO 25) fish oil (80.3% vs. 89.7% and 92.7%, respectively; *p* < 0.05) (Figure 1).

### 3.1. Brain

With regard to the brain, the dietary treatment did not affect the proportions of total SFA (32.9% of the total FA on average), MUFA (29.2%), PUFA (37.9%), n-3 PUFA (29.6%), or n-6 PUFA (8.0%) present in the tissue (*p* > 0.05) (Table 3). Nevertheless, the replacement of FO with VO decreased the relative content of EPA (3.01% vs. 2.81% and 2.85% in FO 100 vs. FO 25 and FO 0, respectively; *p* < 0.001) and DHA (23.8% vs. 21.4% in FO 100 and FO 25 vs. FO 0; *p* < 0.01), whereas it increased the content of ALA (0.81% vs. 1.63% vs. 2.66% in FO 100 vs. FO 25 vs. FO 0; *p* < 0.001).

### 3.2. Liver

In the liver, the replacement of FO with VO in the diet significantly decreased (*p* < 0.001) the proportion of the total SFA (24.3% and 23.3% vs. 20.2% of the total FA in FO 100 and FO 25 vs. FO 0), which was due to the lower proportion of C16:0 in VO than FO (13.9% and 13.4% vs. 11.6%) (Table 4).

Moreover, the substitution of FO with VO in the diet decreased the relative content of C16:1 n-9 (3.26% vs. 2.23% vs. 1.64% in FO 100 vs. FO 25 vs. FO 0) and C17:1 n-7 (7.30% vs. 5.77% vs. 4.95%), whereas it increased that of C18:1 n-9 (32.0% vs. 35.2% vs. 37.6%) (*p* < 0.001). Thus, the proportion of the total MUFA (45.2% on average) was not affected by the dietary treatment (*p* > 0.05). With regard to n-3 PUFA, the inclusion of less FO in the diet decreased the liver tissue’s relative content of EPA (2.21% vs. 1.45% vs. 1.10% in FO 100 vs. FO 25 vs. FO 0), C22:5 n-3 (2.35% vs. 1.35% vs. 0.83%), and DHA (4.26% vs. 2.29% and 1.57%), whereas it greatly increased (*p* < 0.001) its content of ALA (2.48% vs. 8.60% vs. 11.3%), and thus the total n-3 PUFA content. As for n-6 PUFA, the substitution of FO with VO decreased the AA proportion of liver tissue (0.89% vs. 0.53% vs. 0.39%; *p* < 0.001) and increased that of LA (15.2% and 15.2% vs. 17.0% in FO 100 and FO 25 vs. FO 0; *p* < 0.01).

### 3.3. Visceral Fat

In the visceral fat, the total substitution of FO with VO in the diet significantly decreased (*p* < 0.001) the proportion of the total SFA (23.6% and 23.8% vs. 22.5% of the total FA in FO 100 and FO 25 vs. FO 0) (Table 5). Moreover, the replacement of FO with VO decreased the C16:1 n-9 (4.07% vs. 2.98% vs. 2.48% in FO 100 vs. FO 25 vs. FO 0) and 18:1 n-7 (4.89% vs. 4.11% vs. 3.64%) proportions in the visceral fat, whereas it increased that of C18:1 n-9 (27.8% vs. 30.0% vs. 32.6%) (*p* < 0.001). With regard to n-3 PUFA, the inclusion of less FO in the diet decreased the EPA (3.68% vs. 2.76% vs. 2.07%), C22:5 n-3 (1.82% vs. 1.24% vs. 0.9%), and DHA (9.57% vs. 5.72% vs. 4.01%) proportions in the visceral fat, and increased that of ALA therein (3.76% vs. 9.44% vs. 12.0%) (*p* < 0.001), whereas the proportion of total n-3 PUFA (19.2% on average) was not affected by diet (*p* > 0.05). As for n-6 PUFA, the substitution of FO with VO increased the LA relative content (14.6% vs. 16.2% vs. 16.9%), which affected the total proportion of n-6 PUFA in this tissue (17.4% vs. 17.9% vs. 18.2%) (*p* < 0.001).

### 3.4. Muscle

In the red muscle, the replacement of FO with VO did not affect the proportion of total SFA (24.0% of the total FA on average) (Table 6). Among MUFA, the replacement of FO with VO increased the relative content of C18:1 n-9 (27.2% vs. 29.0% vs. 31.8% in FO 100 vs. FO 25 vs. FO 0), whereas it decreased that of C18:1 n-7 (4.21% vs. 3.65% vs. 3.26% in FO 100 vs. FO 25 vs. FO 0) and C20:1 n-9 (1.91% vs. 1.05% vs. 0.74%) (*p* < 0.001). For n-3 PUFA, the inclusion of less FO in the diet decreased the proportions of EPA (3.22% vs. 2.60% vs. 2.14%), C22:5 n-3 (2.12% vs. 1.58% vs. 1.29%), and DHA (14.0% vs. 9.38% vs. 7.47%) in red muscle tissue, whereas it increased that of ALA (2.99% vs. 7.68% vs. 9.84%) (*p* < 0.001). In addition, the partial and total substitution of FO with VO in the diet increased the total n-6 PUFA content of red muscle (14.7% vs. 16.0% and 16.7%) due to changes in the proportions of LA (*p* < 0.001).

In the white muscle, the substitution of FO with VO in the diet decreased the proportion of total SFA (24.0% and 23.8% vs. 22.6% in FO 100 vs. FO 25 vs. FO 0) and increased that of C18:1 n-9 (27.6% vs. 29.0% vs. 31.7%); this also decreased the relative content of C16:1 n-9 (3.78% vs. 2.90% vs. 2.36%) and C20:1 n-9 (1.76% vs. 1.09% vs. 0.76%) in white muscle tissue (Table 7). For n-3 PUFA, the inclusion of less FO in the diet decreased the EPA (3.41% vs. 2.87% vs. 2.26%), C22:5 n-3 (1.71% vs. 1.38% vs. 1.05%), and DHA (11.6% vs. 8.59% vs. 6.38%) content in white muscle tissue, whereas it increased the ALA content (3.76% vs. 9.44% vs. 12.0%) (*p* < 0.001). Nevertheless, the proportion of total n-3 PUFA (21.0% on average) was not affected (*p* > 0.05) by dietary treatment. Finally, the LA relative content in the white muscle was increased by this treatment (14.2% vs. 15.6% and 16.5%), which affected the total n-6 PUFA content (15.6% vs. 16.7% vs. 17.3%) (*p* < 0.001).

## 4. Discussion

Successful growth performance may be achieved in fish fed with diets containing low levels of FO as long as their minimum EFA requirements are met [39]. In the larval stages, DHA plays a key role in promoting the development of neural membranes and eyes [40], improving growth, vitality, and survival [1,14,41], and preventing swimming disorders such as spinning and disorientation [41]. The accumulation of DHA in the brain during fish development has been measured in several marine species [14], which also show very low rates of DHA biosynthesis. Thus, any DHA deficiency during larval development will have serious consequences for the successful performance of fish larvae [14]. Moreover, DHA is essential for the development of schooling behaviour in the Mediterranean yellowtail [42,43,44]. Data in the published literature show that larvae (aged 3–7 days after hatching (dah)) of *Seriola dumerili* fed with rotifers [1] require a supply of DAH equal to 1.5% of the dry matter (DM) in their diet. Larvae (7 mm in length) of the related Japanese amberjack (*S. quinqueradiata* Temminck and Schlegel, 1845) fed with *Artemia* required a diet containing 3.9% of n-3 HUFA (2.5% and 1.3% DM of EPA and DHA, respectively) in a previous study [41]. Further, in *S. rivoliana* Valenciennes, 1833 larvae (aged 30 to 50 dah) had higher survival rates and better stress resistance when fed microdiets containing 3.2% DM of DHA [45].

In this experiment, the dietary treatments tested provided a minimum n-3 HUFA supply of 0.75% DM, which should be sufficient to meet the EFA requirements of most marine species (minimum value: 0.5% DM in juveniles and subadults of marine species [39]). Under different conditions, the total EFA requirements for juveniles (39 to 387 g of live weight in a 154-day feeding trial) of Mediterranean yellowtail have been estimated to be 1.2% DM [11]. In fact, juvenile fish, although they still need EFA, are likely to require lower levels of EPA and DHA in their diet than those needed in the larval stage. Nevertheless, dietary DHA deprivation in juveniles of pelagic marine species, such as carangids and tunids, can be particularly deleterious because of their fast growth rates [14], especially when they are fed diets containing low levels of FO.

During the grow-out phase, EFA deficiencies may reduce fish growth. In this regard, a substitution of 60% of the FO with VO in the diet is considered acceptable [46]. For carangid species, the growth performance of Japanese yellowtail (*S. quinqueradiata*) (average initial weight: 252 g; final weight 412 g) was not affected by the full replacement of FO with olive oil in a previous short-term feeding trial (40 days) [47]. On the contrary, yellowtail kingfish (*S. lalandi* Valenciennes, 1833) showed impaired growth rates when FO was totally substituted with canola or sunflower oil in their diet during a five-week-long feeding trial (with a weight change from 96 to 260 g) [47].

In the present study of *S. dumerili* (grown from 176 to 417 g of live weight, with 109 days of feeding), diets containing only VO as the lipid source had no detrimental impacts on the growth performance of these fish. Nevertheless, the survival rate was lower in fish fed diets without fish oil compared with those in fish fed diets containing 100% (FO 100) or 25% (FO 25) fish oil.

Indeed, Monge-Ortiz et al. [11] reported that diets including at least 525 g kg^−1^ of fish meal (FM) are likely to supply enough LC-PUFA to meet the needs of fish, even with the complete substitution of FO (with fish reaching a final weight of 397 g). In fact, FM usually contains up to 8%–15% DM of crude lipid, 30%–35% of which is composed of n-3 LC-PUFA [19]. In the present study, the experimental diets contained 350 g kg^−1^ of FM, which likely met the FA requirements of the studied fish during the grow-out phase. 

The present study measured fat storage in different tissues, including the muscles, brain, liver, and visceral fat. Indeed, the locations of fat storage differ within and between fish species [15,48,49]. In a previous study [50], a high lipid content (53% DM) was found in the liver of farmed *S. dumerili*, which was consistent with that recorded in the present study (60% DM) and higher than the fat content of the muscle tissue (12% DM) [50].

The replacement of FO with VO in fish diets may lead to the accumulation of fat in the fish liver, generating a fatty liver syndrome, which may be the result of inefficient nutrient utilisation and increased rates of lipid peroxidation [51,52]. Nevertheless, we did not find differences in the liver fat content of the tested fish, even when they were fed with diets containing only VO as the lipid source, which was consistent with the findings of previous studies done on European seabass [53], gilthead sea bream [54], and turbot (*Psetta maxima* (L.; 1758)) [55].

Regardless of whether FO replacement affects fish growth and the lipid content of their tissues, its impact on fatty acid composition will vary depending on the dietary lipid content and source, as well as on the tissues considered [19]. The fatty acid signatures of fish tissues are closely related to their dietary FA composition [19]. Complete or partial FO replacement with VO blends is known to affect the FA compositions of muscles, fish organs, and fat storage tissues [19]. However, the magnitude of the changes in FA signatures varies among different fish species and tissues [56,57,58].

The FA signature of several wild and farmed fish was previously measured in muscle and liver tissues, but to our knowledge, few studies have investigated the FA signatures of other fatty tissues, such as those of the visceral fat and brain. Specifically, in wild and farmed Mediterranean yellowtail, the FA composition has been analysed in the muscles, liver, ovary [10,11,50,59,60], and eggs [61]. Other studies have been performed in other *Seriola* species, such as *S. lalandi* [47,62,63], *S. quinqueradiata* [64,65], *S. dorsalis* (Gill, 1863) [66,67], and *S. rivoliana* [45], which mainly focused on the FA composition of muscle tissues.

In the liver of Mediterranean yellowtail, we found a high proportion of MUFA and a low proportion of SFA, likely because their C16:0, C18:0, and C22:0 content was sufficient to maintain or even exceed the requirements of liver metabolic functions. In the brain and visceral fat, PUFA were highly represented (representing on average 37.9% and 37.1% of the total FA content in the brain and visceral fat, respectively). In fact, PUFA (especially C18 FA, such as LA and ALA) are generally stored in non-lipogenic tissues, especially the visceral fat, and are likely used as metabolic substrates for β-oxidation [58,68,69]. Moreover, the brain contained a high proportion of DHA (on average 23.0% of the total FA), which is known to regulate membrane fluidity, the blood-brain barrier, and the activities of certain enzymes, such as ionic channel proteins and nerve growth factors, both in mammals [70,71] and fish [1,14,45]. In salmonids, Atlantic cod, and flatfishes, DHA is also present at high levels in the brain [12,15,72], which agrees with our findings.

Consistent with other studies [56,73,74], the muscle, liver, and visceral fat FA signatures of Mediterranean yellowtail found in our study reflected the dietary FA profiles of these fish, with the FA signatures of these tissues thus showing a low robustness to changes in the dietary lipid sources from FO to VO. In contrast, the brain FA signature was less strongly affected by the dietary FA composition, whereas only ALA, EPA, and DHA changed. Nevertheless, in European seabass, brain lipids appeared to be sensitive to dietary lipid inputs [75]. On the contrary, a more recent study in juvenile European seabass fed n-3 LC-PUFA-deficient diets showed that polar lipids in their neural tissues had the highest capacity to regulate and preserve their DHA content [76]. In the present study, fish fed the diet with 100% VO showed a 10% lower DHA content in the brain than those fed diets with fish oil (FO 100 and FO 25) and a lower survival, which could have been related to some effects of EFA deficiency in them. In contrast, the administration of the FO 25 diet did not decrease the DHA proportion in the brain, which suggests that the substitution of 75% of the FO in the diet with VO could be suitable for use in diets for rearing Mediterranean yellowtail juveniles from 176 to 417 g of live weight. Further studies are necessary to confirm these results over longer feeding periods.

## 5. Conclusions

This study provided new findings on the FA signatures in different tissues of *Seriola dumerili* fed diets with decreasing levels of FO included in them. The FA composition of the diet had a strong effect on the FA compositions of muscle, liver, and visceral fat tissues. The brain had a FA signature that was more robust to dietary changes, whereas only some EFA (EPA and DHA) decreased when fish oil was totally replaced by vegetable oil.

## Figures and Tables

**Table 1:** Ingredients (g kg^−1^ as fed) and proximate composition (% dry matter; DM) of the experimental diets.

	Diet		
	FO 100	FO 25	FO 0
Fish meal	350	350	350
Wheat	100	100	100
Wheat gluten	140	140	140
Defatted soybean meal	185	185	185
Iberian meat meal	110	110	110
Fish oil	95	24	0
Linseed oil	-	28	38
Sunflower oil	-	21	28
Palm oil	-	22	29
Multivitamin and minerals mix	20	20	20
Proximate composition			
Dry matter, %	87.4	88.8	89.1
Crude protein, % DM	58.8	60.6	58.8
Crude lipid, % DM	15.9	15.1	16.6
Ash, %DM	8.4	10.3	8.3
Gross energy, MJ kg^−1^ DM	24.3	23.8	24.4

Fish oil (FO) 100 diet: Diet formulated with fish oil as lipid source; FO 25 diet: Diet in which fish oil was included at a content of 25%; FO 0 diet: Diet in which fish oil was totally substituted with vegetable oil. ^1^ Vitamins and mineral mixture (values are g kg^−1^): Premix, 25; Hill, 10; DL-α-tocopherol, 5; ascorbic acid, 5; (PO_4_)_2_Ca_3_, 5. Premix composition (values are IU kg^−1^): Retinol acetate, 1,000,000; calcipherol, 500; DL-α-tocopherol, 10; menadione sodium bisulphite, 0.8; hidroclorhidrate thiamine, 2.3; riboflavin, 2.3; pyridoxine hydrochloride, 15; cyanocobalamin, 25; nicotinamide, 15; pantothenic acid, 6; folic acid, 0.65; biotin, 0.07; ascorbic acid, 75; inositol, 15; betaine, 100; polypeptides, 12.

**Table 2:** Fatty acid composition (% of total fatty acid content) of the experimental diets.

	Diet		
	FO 100	FO 25	FO 0
14:0	3.28	2.38	1.65
16:0	18.88	19.58	18.93
17:0	0.53	0.24	0.16
18:0	5.07	4.88	4.71
Σ SFA	27.79	27.12	25.46
16:1 n-9	4.22	2.77	1.82
18:1 n-9	27.14	29.64	32.67
18:1 n-7	3.94	2.94	2.44
22:1 n-9	0.32	0.04	0.06
Σ MUFA	35.62	35.39	36.99
18:2 n-6	12.66	13.36	14.86
18:3 n-6	0.10	0.09	0.09
20:3 n-6	0.10	0.04	0.04
20:4 n-6	1.02	0.58	0.35
22:4 n-6	0.24	0.19	0.09
Σ n-6 PUFA	14.12	14.26	15.43
18:3 n-3	2.24	10.48	14.60
20:3 n-3	0.15	0.08	0.06
20:5 n-3	5.81	4.34	2.77
22:5 n-3	1.29	0.73	0.42
22:6 n-3	12.98	7.61	4.28
Σ n-3 PUFA	22.47	23.24	22.13
Σ PUFA	36.59	37.50	37.56
Σ n-6/Σ n-3	0.63	0.61	0.70
DHA/EPA	2.23	1.76	1.54

FO 100 diet: Diet formulated with fish oil as lipid source; FO 25 diet: Diet in which fish oil was included at a content of 25%; FO 0 diet: Diet in which fish oil was totally substituted with vegetable oil. Abbreviations: SFA: Saturated fatty acids; MUFA: Monounsaturated fatty acids; PUFA: Polyunsaturated fatty acids; DHA/EPA: 22:6 n-3/20:5 n-3.

**Table 3:** Fat content and fatty acid composition (% of total fatty acid content) of the brain in Mediterranean yellowtail fed the experimental diets (*n* = 12 per diet). Values are expressed as least square (LS)means.

	Diet			*p*-Value	RSD
	FO 100	FO 25	FO 0		
Fat, % WW	3.22	4.23	3.52	0.999	0.951
Fatty acids, %					
10:0	0.42	0.46	0.57	0.083	0.138
14:0	0.76	0.64	0.68	0.134	0.141
15:0	0.12 ^b^	0.10 ^a^	0.09 ^a^	<0.001	0.000
16:0	16.31	16.54	16.21	0.378	0.563
17:0	0.19 ^b^	0.14 ^a^	0.14 ^a^	0.006	0.000
18:0	11.42	11.76	11.45	0.367	0.625
20:0	0.46	0.44	0.44	0.475	0.045
22:0	0.79	0.76	0.75	0.618	0.105
24:0	2.41	2.35	2.20	0.387	0.318
Σ SFA	32.90	33.17	32.51	0.278	0.932
16:1 n-9	2.67 ^b^	2.40 ^a^	2.34 ^a^	<0.001	0.114
17:1 n-10	0.55	0.49	0.52	0.824	0.235
18:1 n-9	20.33 ^ab^	19.93 ^a^	21.49 ^b^	0.009	1.050
18:1 n-7	2.80 ^b^	2.58 ^ab^	2.46 ^a^	0.005	0.148
20:1 n-9	0.70 ^b^	0.59 ^a^	0.59 ^a^	<0.001	0.063
22:1 n-9	0.32	0.32	0.28	0.271	0.045
24:1 n-9	2.16	2.07	1.98	0.378	0.261
Σ MUFA	29.47	28.44	29.66	0.139	1.336
18:2 n-6	4.25 ^a^	4.39 ^a^	5.55 ^b^	0.016	1.080
20:2 n-6	0.26	0.23	0.25	0.298	0.055
20:4 n-6	2.65 ^b^	2.51 ^b^	2.27 ^a^	<0.001	0.187
22:2 n-6	0.23 ^b^	0.19 ^ab^	0.18 ^a^	0.028	0.045
22:4 n-6	0.22	0.22	0.18	0.150	0.055
Σ PUFA n-6	7.74	7.69	8.54	0.077	0.967
18:3 n-3	0.81 ^a^	1.63 ^b^	2.66 ^c^	<0.001	0.644
20:3 n-3	0.10 ^a^	0.16 ^b^	0.19 ^b^	<0.001	0.032
20:5 n-3	3.01 ^b^	2.81 ^a^	2.85 ^a^	<0.001	0.118
22:5 n-3	2.13	2.26	2.14	0.483	0.219
22:6 n-3	23.83 ^b^	23.83 ^b^	21.43 ^a^	0.006	1.668
Σ PUFA n-3	29.89	30.71	29.27	0.120	1.127
Σ PUFA	37.63	38.32	37.83	0.176	0.823
Σ n-6/Σ n-3	0.26	0.25	0.30	0.098	0.041
DHA/EPA	7.95 ^ab^	8.50 ^b^	7.54 ^a^	0.023	0.780

FO 100 diet: Diet formulated with fish oil as lipid source; FO 25 diet: Diet in which fish oil was included at a content of 25%; FO 0 diet: Diet in which fish oil was totally substituted with vegetable oil. Abbreviations: WW: Wet weight; RSD: Residual standard deviation; SFA: Saturated fatty acids; MUFA: Monounsaturated fatty acids; PUFA: Polyunsaturated fatty acids; DHA/EPA: 22:6 n-3/20:5 n-3. The FA < 0.1% of the total FA (i.e.; C12:0, C14:1, C18:3 n-6, and C30:3 n-6) are not given in the table. Different superscript letters indicate significant statistical differences among diets (*p* ≤ 0.05).

**Table 4:** Fat content and fatty acid composition (% of total fatty acid content) of the liver in Mediterranean yellowtail fed the experimental diets (*n* = 12 per diet). Values are expressed as LS means.

	Diet			*p*-Value	RSD
	FO 100	FO 25	FO 0		
Fat, % WW	26.6	30.1	25.7	0.721	9.296
Fatty acids, %					
14:0	1.55 ^c^	1.08 ^b^	0.79 ^a^	<0.001	0.129
15:0	0.26 ^c^	0.15 ^b^	0.10 ^a^	<0.001	0.026
16:0	13.88 ^b^	13.40 ^b^	11.57 ^a^	<0.001	0.885
17:0	0.49 ^c^	0.32 ^b^	0.24 ^a^	<0.001	0.037
18:0	7.68 ^b^	7.82 ^b^	7.01 ^a^	0.008	0.621
20:0	0.31 ^c^	0.25 ^b^	0.21 ^a^	<0.001	0.014
24:0	0.06	0.07	0.05	0.369	0.020
Σ SFA	24.32 ^b^	23.26 ^b^	20.07 ^a^	<0.001	1.317
16:1 n-9	3.26 ^c^	2.23 ^b^	1.64 ^a^	<0.001	0.153
17:1 n-10	0.26	0.18	0.19	0.323	0.139
18:1 n-9	32.02 ^a^	35.18 ^b^	37.55 ^c^	<0.001	1.371
18:1 n-7	7.30 ^c^	5.77 ^b^	4.95 ^a^	<0.001	0.289
20:1 n-9	1.88 ^b^	1.17 ^a^	0.90 ^a^	<0.001	0.314
22:1 n-9	0.37 ^b^	0.24 ^a^	0.16 ^a^	<0.001	0.040
24:1 n-9	0.21 ^b^	0.12 ^a^	0.09 ^a^	<0.001	0.063
Σ MUFA	45.29	44.90	45.47	0.613	1.431
18:2 n-6	15.23 ^a^	15.21 ^a^	16.96 ^b^	0.002	1.10
18:3 n-6	0.12	0.11	0.11	0.482	0.026
20:2 n-6	1.23 ^b^	0.95 ^a^	0.88 ^a^	<0.001	0.097
20:3 n-6	0.21 ^c^	0.13 ^b^	0.08 ^a^	<0.001	0.021
20:4 n-6	0.89 ^c^	0.53 ^b^	0.39 ^a^	<0.001	0.097
22:2 n-6	0.79 ^c^	0.42 ^b^	0.26 ^a^	<0.001	0.052
22:4 n-6	0.26 ^b^	0.08 ^a^	0.05 ^a^	<0.001	0.049
Σ PUFA n-6	18.73 ^b^	17.43 ^a^	18.74 ^b^	0.036	1.207
18:3 n-3	2.48 ^a^	8.60 ^b^	11.27 ^c^	<0.001	0.562
20:3 n-3	0.36 ^a^	0.81 ^b^	0.95 ^c^	<0.001	0.117
20:5 n-3	2.21 ^c^	1.45 ^b^	1.10 ^a^	<0.001	0.200
22:5 n-3	2.35 ^c^	1.35 ^b^	0.83 ^a^	<0.001	0.199
22:6 n-3	4.26 ^b^	2.29 ^a^	1.57 ^a^	<0.001	0.607
Σ PUFA n-3	11.66 ^a^	14.45 ^b^	15.72 ^c^	<0.001	0.924
Σ PUFA	30.39 ^a^	31.84 ^a^	34.46 ^b^	<0.001	1.703
Σ n-6/Σ n-3	1.62 ^b^	1.20 ^a^	1.20 ^a^	<0.001	0.113
DHA/EPA	1.93 ^b^	1.59 ^ab^	1.36^a^	<0.001	0.249

FO 100 diet: Diet formulated with fish oil as lipid source; FO 25 diet: Diet in which fish oil was included at a content of 25%; FO 0 diet: Diet in which fish oil was totally substituted with vegetable oil. Abbreviations: WW: Wet weight; RSD: Residual standard deviation; SFA: Saturated fatty acids; MUFA: Monounsaturated fatty acids; PUFA: Polyunsaturated fatty acids; DHA/EPA: 22:6 n-3/20:5 n-3. The FA < 0.1% of the total FA (i.e.; C12:0, C14:1, C22:0, and C24:0) are not given in the table. Different superscript letters indicate significant statistical differences among diets (*p* ≤ 0.05).

**Table 5:** Fat content and fatty acid composition (% of total fatty acid content) of the visceral fat in Mediterranean yellowtails fed the experimental diets (*n* = 12 per diet). Values are expressed as LS means.

	Diet			*p*-Value	RSD
	FO 100	FO 25	FO 0		
Fat, % WW	37.90	36.90	34.91	0.920	9.529
Fatty acids, %					
14:0	2.55 ^c^	1.99 ^b^	1.64	<0.001	0.094
15:0	0.32 ^c^	0.22 ^b^	0.16 ^a^	<0.001	0.018
16:0	14.57 ^a^	15.00 ^b^	14.36 ^a^	<0.001	0.293
17:0	0.44 ^c^	0.32 ^b^	0.26 ^a^	<0.001	0.023
18:0	5.11 ^a^	5.64 ^b^	5.46^b^	<0.001	0.207
20:0	0.36 ^c^	0.32 ^b^	0.29 ^a^	<0.001	0.009
22:0	0.14 ^a^	0.18 ^b^	0.19 ^b^	0.001	0.011
24:0	0.10	0.11	0.12	0.307	0.026
Σ SFA	23.63 ^b^	23.80 ^b^	22.53 ^a^	<0.001	0.415
16:1 n-9	4.07 ^c^	2.98 ^b^	2.48 ^a^	<0.001	0.184
17:1 n-10	0.37 ^b^	0.20 ^a^	0.16 ^a^	<0.001	0.049
18:1 n-9	27.8 ^a^	30.0 ^b^	32.6 ^c^	<0.001	0.547
18:1 n-7	4.89 ^c^	4.11 ^b^	3.64 ^a^	<0.001	0.154
20:1 n-9	1.96 ^c^	1.11 ^b^	0.79 ^a^	<0.001	0.116
22:1 n-9	0.40 ^c^	0.22 ^b^	0.15 ^a^	<0.001	0.046
24:1 n-9	0.49 ^c^	0.30 ^b^	0.23 ^a^	<0.001	0.038
Σ MUFA	39.95 ^b^	38.87 ^a^	40.07 ^b^	<0.001	0.495
18:2 n-6c	14.64 ^a^	16.17 ^b^	16.89 ^c^	<0.001	0.534
18:3 n-6	0.14 ^b^	0.12 ^a^	0.12 ^a^	<0.001	0.003
20:2 n-6	0.92 ^c^	0.64 ^b^	0.48 ^a^	<0.001	0.043
20:3 n-6	0.15 ^b^	0.07 ^ab^	0.06 ^a^	0.023	0.075
20:4 n-6	0.67 ^c^	0.43 ^b^	0.32 ^a^	<0.001	0.044
22:2 n-6	0.52 ^c^	0.31 ^b^	0.26 ^a^	<0.001	0.044
22:4 n-6	0.34 ^c^	0.18 ^b^	0.10 ^a^	<0.001	0.046
Σ PUFA n-6	17.37 ^a^	17.92 ^ab^	18.19 ^b^	0.003	0.534
18:3 n-3	3.76 ^a^	9.44 ^b^	12.0 ^c^	<0.001	0.843
20:3 n-3	0.22 ^a^	0.26 ^b^	0.28 ^b^	<0.001	0.022
20:5 n-3	3.68 ^c^	2.76 ^b^	2.07 ^a^	<0.001	0.150
22:5 n-3	1.82 ^c^	1.24 ^b^	0.90 ^a^	<0.001	0.010
22:6 n-3	9.57 ^c^	5.72 ^b^	4.01 ^a^	<0.001	0.634
Σ PUFA n-3	19.05	19.40	19.21	0.266	0.372
Σ PUFA	36.42 ^a^	37.33 ^b^	37.40 ^b^	0.002	0.613
Σ n-6/Σ n-3	0.91	0.92	0.95	0.104	0.035
DHA/EPA	2.60 ^b^	2.07 ^a^	1.94 ^a^	<0.001	0.153

FO 100 diet: Diet formulated with fish oil as lipid source; FO 25 diet: Diet in which fish oil was included at a content of 25%; FO 0 diet: Diet in which fish oil was totally substituted with vegetable oil. Abbreviations: WW: Wet weight; RSD: Residual standard deviation; SFA: Saturated fatty acids; MUFA: Monounsaturated fatty acids; PUFA: Polyunsaturated fatty acids; DHA/EPA: 22:6 n-3/20:5 n-3. The FA < 0.1% of the total FA (i.e.; C10:0, C12:0, C14:1) are not given in the table. Different superscript letters indicate significant statistical differences among diets (*p* ≤ 0.05).

**Table 6:** Fat content and fatty acid composition (% of total fatty acid content) of the red muscle in Mediterranean yellowtails fed the experimental diets (*n* = 12 per diet). Values are expressed as LS means.

	Diet			*p*-Value	RSD
	FO 100	FO 25	FO 0		
Fat, % WW	4.37	4.89	4.49	0.500	0.816
Fatty acids, %					
14:0	1.81	1.81	1.26	0.105	0.602
16:0	14.41	15.01	14.40	0.125	0.455
17:0	0.44 ^c^	0.30 ^b^	0.24 ^a^	<0.001	0.035
18:0	6.53 ^a^	6.91 ^b^	6.83 ^b^	<0.001	0.184
20:0	0.38 ^b^	0.33 ^a^	0.32 ^a^	<0.001	0.017
22:0	0.12 ^a^	0.15 ^b^	0.16 ^b^	<0.001	0.015
Σ SFA	23.88	24.65	23.33	0.061	0.769
14:1 n-9	0.11	0.12	0.10	0.956	0.100
16:1 n-9	3.14 ^b^	2.57 ^ab^	2.05 ^a^	0.005	0.125
17:1 n-10	0.31 ^b^	0.21 ^a^	0.16 ^a^	<0.001	0.011
18:1 n-9	27.21 ^a^	29.04 ^b^	31.76 ^c^	<0.001	0.509
18:1 n-7	4.21 ^c^	3.65 ^b^	3.26 ^a^	<0.001	0.208
20:1 n-9	1.91 ^c^	1.05 ^b^	0.74 ^a^	<0.001	0.134
22:1 n-9	0.36 ^b^	0.18 ^a^	0.13 ^a^	<0.001	0.088
24:1 n-9	0.41 ^c^	0.26 ^b^	0.20 ^a^	<0.001	0.038
Σ MUFA	37.62 ^ab^	37.08 ^a^	38.37 ^b^	0.044	0.651
18:2 n-6c	13.05 ^a^	14.83 ^b^	15.76 ^c^	<0.001	0.552
18:3 n-6	0.12	0.11	0.10	0.053	0.015
20:2 n-6	0.78 ^b^	0.58 ^a^	0.45 ^a^	<0.001	0.027
20:4 n-6	0.91 ^c^	0.65 ^b^	0.54 ^a^	<0.001	0.055
22:2 n-6	0.40 ^c^	0.24 ^b^	0.16 ^a^	<0.001	0.031
22:4 n-6	0.51 ^c^	0.29 ^b^	0.21 ^a^	<0.001	0.040
Σ PUFA n-6	14.70 ^a^	15.95 ^b^	16.68 ^b^	<0.001	0.513
18:3 n-3	2.99 ^a^	7.68 ^b^	9.84 ^c^	<0.001	0.705
20:3 n-3	0.20 ^a^	0.25 ^b^	0.27 ^b^	<0.001	0.021
20:5 n-3	3.22 ^c^	2.60 ^b^	2.14 ^a^	<0.001	0.116
22:5 n-3	2.12 ^c^	1.58 ^b^	1.29 ^a^	<0.001	0.155
22:6 n-3	14.00 ^c^	9.38 ^b^	7.47 ^a^	<0.001	0.946
Σ PUFA n-3	22.56 ^b^	21.50 ^ab^	21.01 ^a^	0.036	0.884
Σ PUFA	38.50	38.27	38.30	0.925	0.881
Σ n-6/Σ n-3	1.54 ^b^	1.35 ^a^	1.26 ^a^	<0.001	0.083
DHA/EPA	4.34 ^b^	3.61 ^a^	3.49 ^a^	0.001	0.280

FO 100 diet: Diet formulated with fish oil as lipid source; FO 25 diet: Diet in which fish oil was included at a content of 25%; FO 0 diet: Diet in which fish oil was totally substituted with vegetable oil. Abbreviations: WW: Wet weight; RSD: Residual standard deviation; SFA: Saturated fatty acids; MUFA: Monounsaturated fatty acids; PUFA: Polyunsaturated fatty acids; DHA/EPA: 22:6 n-3/20:5 n-3. The FA < 0.1% of the total FA (i.e.; C12:0, C15:0, C20:3 n-6, and C24:0) are not given in the table. Different superscript letters indicate significant statistical differences among diets (*p* ≤ 0.05).

**Table 7:** Fat content and fatty acid composition (% of total fatty acid content) of the white muscle in Mediterranean yellowtails fed the experimental diets (*n* = 12 per diet). Values are expressed as LS means.

	Diet			*p*-Value	RSD
	FO 100	FO 25	FO 0		
Fat, % WW	5.84	5.94	6.03	0.941	0.770
Fatty acids, %					
14:0	2.28 ^c^	1.80 ^b^	1.47 ^a^	<0.001	0.132
16:0	14.97 ^b^	14.78 ^b^	14.22 ^a^	0.035	0.366
17:0	0.39 ^c^	0.30 ^b^	0.23 ^a^	<0.001	0.037
18:0	5.89	6.21	6.13	0.050	0.171
20:0	0.36 ^c^	0.33 ^b^	0.32 ^a^	<0.001	0.013
22:0	0.12 ^a^	0.15 ^b^	0.16 ^b^	0.020	0.032
Σ SFA	23.94 ^b^	23.80 ^b^	22.63 ^a^	0.009	0.494
14:1 n-9	0.28 ^c^	0.22 ^b^	0.15 ^a^	<0.001	0.038
16:1 n-9	3.78 ^c^	2.90 ^b^	2.36 ^a^	<0.001	0.200
17:1 n-10	0.33 ^c^	0.22 ^b^	0.17 ^a^	<0.001	0.026
18:1 n-9	27.55 ^a^	29.02 ^b^	31.72 ^c^	<0.001	0.759
18:1 n-7	4.15 ^c^	3.56 ^b^	3.20 ^a^	<0.001	0.165
20:1 n-9	1.76 ^c^	1.09 ^b^	0.76 ^a^	<0.001	0.175
22:1 n-9	0.29 ^c^	0.19 ^b^	0.12 ^a^	<0.001	0.045
24:1 n-9	0.35 ^c^	0.27 ^b^	0.19 ^a^	<0.001	0.048
Σ MUFA	38.63	37.50	38.63	0.050	0.615
18:2 n-6c	14.15 ^a^	15.59 ^b^	16.46 ^b^	<0.001	0.552
18:3 n-6	0.14 ^b^	0.13 ^a^	0.12 ^a^	0.001	0.009
20:2 n-6	0.86 ^c^	0.63 ^b^	0.49 ^a^	<0.001	0.059
20:4 n-6	0.79 ^c^	0.61 ^b^	0.48 ^a^	<0.001	0.076
22:2 n-6	0.42 ^c^	0.28 ^b^	0.19 ^a^	<0.001	0.037
22:4 n-6	0.41 ^c^	0.26 ^b^	0.18 ^a^	<0.001	0.052
Σ PUFA n-6	16.45 ^a^	17.20 ^ab^	17.80 ^b^	0.001	0.493
18:3 n-3	3.93 ^a^	8.25 ^b^	10.84 ^c^	<0.001	1.141
20:3 n-3	0.20 ^a^	0.23 ^b^	0.25 ^c^	<0.001	0.019
20:5 n-3	3.41 ^c^	2.87 ^b^	2.26 ^a^	<0.001	0.198
22:5 n-3	1.71 ^c^	1.38 ^b^	1.05 ^a^	<0.001	0.136
22:6 n-3	11.63 ^c^	8.59 ^b^	6.38 ^a^	<0.001	1.196
Σ PUFA n-3	20.60	21.21	20.77	0.830	0.773
Σ PUFA	37.48	38.70	38.74	0.500	0.825
Σ n-6/Σ n-3	0.80	0.81	0.86	0.086	0.069
DHA/EPA	3.43 ^b^	2.95 ^a^	2.82 ^a^	0.019	0.234

FO 100 diet: Diet formulated with fish oil as lipid source; FO 25 diet: Diet in which fish oil was included at a content of 25%; FO 0 diet: Diet in which fish oil was totally substituted with vegetable oil. Abbreviations: WW: Wet weight; RSD: Residual standard deviation; SFA: Saturated fatty acids; MUFA: Monounsaturated fatty acids; PUFA: Polyunsaturated fatty acids; DHA/EPA: 22:6 n-3/20:5 n-3. The FA < 0.1% of the total FA (i.e.; C12:0, C15:0, C20:3 n-6, and C24:0) are not given in the table. Different superscript letters indicate significant statistical differences among diets (*p* ≤ 0.05).
